# Determination of Hydroquinone Clathrate Stability for Different Guest Molecules Using *NPH* Ensemble Molecular Dynamics

**DOI:** 10.1021/acs.energyfuels.6c02939

**Published:** 2026-09-30

**Authors:** Brais R. García, Jesús Algaba, Martín Pérez-Rodríguez, María M. Conde, Felipe J. Blas, Manuel M. Piñeiro

**Affiliations:** † Departamento de Física Aplicada, 16784 Universidade de Vigo, Vigo 36310, Spain; ‡ Laboratorio de Simulación Molecular y Química Computacional, CIQSO-Centro de Investigación en Química Sostenible and Departamento de Ciencias Integradas, 16743 Universidad de Huelva, Huelva 21006, Spain; § Instituto de Química Física Blas Cabrera, CSIC, Madrid E-28006, Spain; ∥ Departamento de Ingeniería Química Industrial y del Medio Ambiente, Escuela Técnica Superior de Ingenieros Industriales, 16771 Universidad Politécnica de Madrid, Madrid 28006, Spain

## Abstract

Clathrates are crystalline compounds with a wide range of applications, particularly in the fields of storage of substances of interest (such as hydrogen) and gas separation. In this work, molecular dynamics simulations are used to investigate the stability of *β*-hydroquinone clathrates (HQ clathrates) containing guest molecules such as Ar, CH_4_, H_2_, and CO_2_ in a pressure range between 100 and 1000 bar. To this end, the isobaric-isenthalpic (*NPH*) ensemble was used, which allows the equilibrium conditions to be studied without applying artificial temperature control, providing a direct and more realistic view of the thermodynamic response of the system. Atomistic models based on the OPLS-AA force field are used for this purpose. The results reveal a tendency for the coexistence temperature to increase with pressure, which is more pronounced for larger, apolar guests (Ar and CH_4_) compared to smaller, polar species (H_2_, CO_2_). This study represents the first application of the *NPH*-based methodology to organic clathrates, demonstrating its effectiveness in studying the limiting thermodynamic conditions associated with phase transitions. The findings presented provide a solid theoretical framework for understanding the dynamic behavior of HQ clathrates and open up new perspectives for their use in the safe and reversible storage of hydrogen.

## Graphical abstract

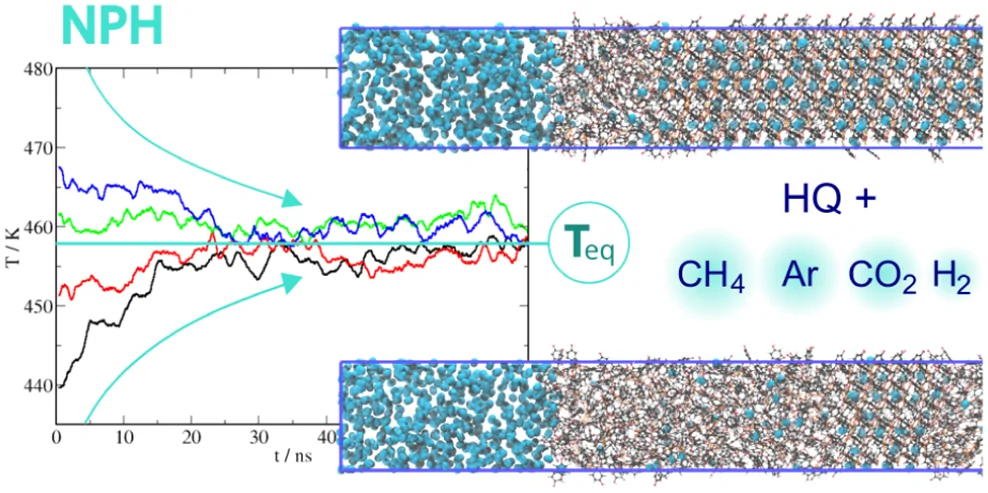

## Introduction

In the late 18th century, during early studies of the solubility of newly discovered “airs” (gases), several natural philosophers reported the unexpected appearance of ice-like solids – even at temperatures above the normal freezing point of water – when certain gases were bubbled through cold water or when such mixtures were frozen. Sir Humphry Davy, a pioneer of electrochemistry and mentor to Michael Faraday, later recognized these materials as compounds formed by water and gas and introduced the term “gas hydrates”.^1,2^ In 1810, Davy discovered chlorine hydrate by cooling a water solution saturated with chlorine to temperatures below 9^◦^ C. Subsequent studies by Villard, de Forcrand, and many others showed that hydrates of this kind can form when water is combined with a wide range of gases or volatile liquids, including chlorine (Cl_2_), bromine (Br_2_), hydrogen sulfide (H_2_S), carbon dioxide (CO_2_), methane (CH_4_), trichloromethane (CHCl_3_), as well as the noble gases argon (Ar), krypton (Kr), and xenon (Xe).

By the 1950s, more than a century after the discovery of hydroquinone (HQ) by Wöhler,^3^ Palin and Powell^4,5^ and subsequently Powell^6,7^ examined a series of unusual inclusion compounds formed by HQ or benzene-1,4-diol, with formula C_6_H_6_O_2_, with a wide range of gases and volatile liquids. HQ is an aromatic phenolic compound (a para-dihydroxy derivative of benzene) bearing two hydroxyl groups in the 1,4-positions of the ring. Their studies showed that HQ molecules self-assemble via hydrogen bonding into two interpenetrating three-dimensional networks that generate approximately spherical cavities, corresponding to a stoichiometry of about one cavity per three HQ molecules.

HQ crystallizes in several polymorphs, commonly denoted *α*-, *β*-, *γ*-, and *δ*-HQ. The *α* polymorph is the thermodynamically stable form of pure HQ under ambient conditions.^8^ The monoclinic *γ* phase can be obtained by sublimation or by rapid evaporation of HQ solutions in ether.^9^ Among these polymorphs, clathrate formation in the presence of suitable guests is observed only for *β*-HQ.^10^ Notably, empty *β*-HQ is metastable: only partial filling of the cavities by guest molecules stabilizes the framework over a finite range of temperatures and pressures. Finally, the *δ* phase, which is accessed in pure HQ at elevated pressure and temperature, has been reported only recently.^11^

Over the subsequent decades of research, gas hydrates were recognized as clathrates: crystalline inclusion compounds in which small guest molecules are trapped within a hydrogen-bonded host framework. Canonical examples include CH_4_ and CO_2_ clathrates under suitable thermodynamic conditions. When the hydrogen-bonded host lattice is formed by water, these materials are termed clathrate hydrates (or simply hydrates), as in Davy’s original chlorine hydrate. More generally, the host framework can be provided by other hydrogen-bond-forming molecules – often organic species – capable of assembling into three-dimensional crystalline networks. Several substances can form clathrates. One notable example is HQ, but other organic molecules can also act as hosts in these materials, including urea and Dianin’s compound, among many others.^12–16^

Clathrate frameworks have also been reported in intermetallic systems,^17,18^ where metallic guest ions are encapsulated by cages built from group 14 elements (Si, Ge, or Sn). More recently, clathrate-like architectures have been shown to self-assemble from colloidal building blocks,^19,20^ enabling larger cage sizes and mesoscale phenomena with potential relevance to biological and photonic applications.

A key common point between traditional gas hydrates and more challenging and sophisticated clathrates is the host–guest interaction, which underpins their use in natural gas capture^1^ and gas storage.^21^ CH_4_ hydrates, in particular, have garnered significant attention as an alternative energy resource due to their vast natural deposits.^1,2,22–29^ Additionally, hydrates play a critical role in climate dynamics, as they represent a major reservoir of greenhouse gases that could be destabilized under changing environmental conditions.^1,2,22,23,30–32^ Beyond energy and climate, gas hydrates are also being explored for their potential in CO_2_ sequestration,^33–35^ as well as for gas storage^36^ and transportation technologies.^37–39^

In the case of HQ clathrates, the continued interest for them is largely driven by their potential as selective solid hosts for compounds of industrial or strategic relevance. For instance, HQ clathrates have been considered as materials for (hydrogen) H_2_ storage.^40,41^ Lee and coworkers have explored the controlled separation of gas mixtures using clathrates,^42–45^ reporting a striking preference of HQ clathrates for CO_2_. This selectivity motivates their potential use as CO_2_ sorbents/scrubbers^46^ in decarbonization-related processes. A comprehensive account of structural and spectroscopic features, phase equilibria, dynamics, cage occupancy, and CH_4_/CO_2_ selectivity for HQ clathrates is available in a dedicated series of studies by Torré and coworkers.^47–53^

As we have already mentioned, one of the most studied systems is based on HQ, specifically in its *β* crystalline phase^40,42,54–56^ due to its ability to contain a wide variety of guests. Within the *β* phase, it is possible to identify three different structures according to their symmetry, called types I, II, and III, whose formation depends on the nature of the guest occupying the matrix.^6,7,57^ Note that these three types of structures are only compatible with the *β*-HQ polymorph. Species with spherical or quasi-spherical symmetry, such as noble gases, give rise to the type I structure, which belongs to the R3̅ space group.^58,59^ The flexibility and size of the crystal lattice allow the incorporation of large species, such as Ar, hydrogen (H_2_) or CH_4_, although their diffusion becomes less relevant as the atomic radius increases. The type II structure belongs to the R3 group and accommodates molecules such as methanol (CH_3_OH), CO_2_ or hydrochloric acid (HCl). Finally, the type III structure corresponds to the P3 group and appears when the crystal lattice accommodates complex molecules, such as acetonitrile.^57,58,59^ Regardless of the type, it is estimated that the *β* phase has a host–guest ratio of 1:3 under single-occupancy conditions, i.e., one guest per cavity. The *α* phase, for example, has a ratio of 1:18, while the *γ* phase cannot accommodate guests in its crystalline structure, so it is not considered a clathrate. Therefore, the *β* phase is considered to be the most relevant phase in terms of usefulness, and it has been chosen for this work.

This *β* polymorph is made up of channels stacked along a common axis, which allow the diffusion of guests along a preferred direction that facilitates translational movement.^42,55,60^ In relation to the host structure of *β*-HQ, this forms a flexible network capable of adapting the size of its cavities to species of different sizes without the need for a phase change,^42,55,60^ The effective cavity radius of the structure is estimated to be approximately 2.5 Å^61^ which allows it to be classified as an ultramicroporous material. This could enable reversible loading and unloading of the clathrate through a reversible process without the need for melting or recrystallization, unlike hydrates, in which the guests have a much lower diffusion coefficient.^42,55,60,62^ In terms of maximum capacity, it is estimated that *β*-HQ could accommodate up to 4.5 H_2_ molecules per cell;^63^ however, this limit is ideal. Monte Carlo simulations show that the amount of H_2_ incorporated is low under ambient conditions, but increases significantly with increasing pressure or decreasing temperature.^64,65^ Thus, obtaining an accurate theoretical estimation of the clathrate stability limit conditions for HQ-based systems is essential to explore their potential use in practical applications related to gas separation or storage.

According to Gibbs’ phase rule, two-component systems involving solid and liquid phases may display complex phase equilibria and it is not easy to establish the stability limit conditions of these materials. A property of central practical and theoretical relevance is the location of the clathrate three-phase line. For a binary system in which three phases coexist, the number of degrees of freedom is one; consequently, in a three-dimensional representation spanned by pressure, temperature, and mixture composition (e.g., the mole fraction of one component), three-phase equilibrium is mapped onto a one-dimensional curve. Of particular interest is the set of states where the clathrate solid coexists either with two liquid phases or with a liquid and a vapor phase. This curve is commonly referred to as the dissociation line, since it separates a high-temperature region where only fluid and/or vapor phases are stable from a low-temperature region where fluid phases coexist with the crystalline clathrate. An analogous dissociation line is well established for clathrate hydrates. In fact, there is a huge amount of experimental information on the dissociation line of hydrates of different gases, including CH_4_, CO_2_, (nitrogen) N_2_, H_2_, H_2_S, and light hydrocarbons and their mixtures, among many other gases of practical importance.^1^ Unfortunately, the experimental information for dissociation lines of clathrates, in general, and for HQ clathrate, in particular, is really scarce. Only some data for HQ clathrate of some noble gases are available in the literature.^66–71^ Given the current state of the literature, we aim to determine, using atomistic molecular simulations, the coexistence (dissociation) temperatures of HQ clathrates as a function of external pressure for different guest species.

Computer simulation and molecular-based theories provide an alternative, yet complementary, route for predicting thermodynamic properties, including phase equilibria, of clathrate hydrates. In particular, hydrate dissociation lines can be estimated via simulation and molecular models, including frameworks based on the van der Waals–Platteeuw theory. Accordingly, a substantial body of work over the past years has focused on determining hydrate dissociation boundaries using these approaches. By contrast, to the best of our knowledge, the dissociation line of non-aqueous clathrates has not yet been reported from direct computer simulation.

Restricting attention to fully simulation-based strategies, hydrate dissociation lines are most commonly determined using two approaches: direct-coexistence (DC) simulations and the solubility method. The DC approach traces back to the pioneering ideas of Ladd and Woodcock developed in the 1970s.^72^ Conde and Vega later reported the first application of this technique to compute the three-phase coexistence line of methane hydrate,^73,74^ and it has since been adopted by numerous authors to determine dissociation lines for a variety of hydrate formers.^75–88^ In the direct coexistence approach for locating the three-phase (dissociation) line of hydrates, the hydrate phase, the aqueous phase, and a guest reservoir are placed in mutual contact through planar interfaces. The solubility method,^89^ in contrast, relies on calculating the solubility of the guest in an aqueous phase that is simultaneously in equilibrium – through planar interfaces – with a guest-rich liquid phase and with the hydrate. A closely related formulation, in which solubilities are inferred from the solute excess chemical potential in the aqueous phase, was first introduced by Tanaka and coworkers.^90^ Notably, this framework parallels standard procedures used in classical equations of state to locate three-phase coexistence involving gas and/or solid phases.^91–93^ More recently, some of us extended this methodology by computing solubilities directly from molecular simulations, showing that the solubility route provides a distinct yet fully equivalent alternative to the DC approach for determining hydrate dissociation lines.^87,89,94–97^

Computer simulations dealing with planar interfaces using either the direct coexistence technique or the solubility method are usually performed in the *NPT* or isothermal-isobaric ensemble.^98,99^ Strictly speaking, the $NP_{z}AT$ is the correct ensemble for simulations of inhomogeneous systems since the fixed pressure *P*_*z*_ is the equilibrium pressure.^100,101^ This means that only the simulation box length along the *z*-axis is allowed to fluctuate, whereas the box lengths along the *x*- and *y*-axis are kept fixed. This condition must be relaxed when one of the two phases is a solid phase to avoid the simulation of a stressed solid. It should be noted that the use of an isothermal-isobaric ensemble for sampling the system involves fixing two thermodynamic variables, the pressure and temperature. However, according to the Gibbs’ rule phase, only one thermodynamic variable can be fixed when studying a three-phase dissociation line of a hydrate. Due to this, the routinary determination of the dissociation temperature at a given pressure at the three-phase line involves several simulations.

Unfortunately, preliminary prospective calculations performed by our group for the case of HQ clathrates indicate that the CD and SM approaches, when used in combination with constant-*NPT* simulations, are not reliable for tracing the dissociation line, in sharp contrast with their proven performance for classical gas hydrates.

The major obstacle in this case is the limited structural stability of the *β*-HQ phase. Its crystalline network contains channels that may propagate along the reaction coordinate even when no additional guest molecules are present in the surrounding medium. Guests initially trapped within the crystal can diffuse and eventually escape through these channels. During this migration, the guest induces a local reorganization of the host lattice, transiently stabilizing nearby HQ molecules and effectively promoting further channel growth. As a result, the channel can extend without bound while its interior becomes progressively depleted of guest molecules. In practice, this spontaneous evolution of the internal crystal morphology generates an emergent fourth phase – an empty clathrate – within a system that would otherwise be treated as strictly three-phase. Such behavior compromises the stability of coexistence simulations and underscores the need for alternative methodological strategies for systems exhibiting strong reorganization dynamics.

Other statistical ensembles are, *a priori*, better suited to determine dissociation temperatures in hydrates and clathrates than the *NPT* ensemble. As noted above, a constant-*NPT* simulation with three phases in contact will slowly evolve toward a two-phase state, depending on whether the chosen (*P*, *T*) point lies above or below the dissociation line. By contrast, in the isobaric–isenthalpic (*NPH*) ensemble the two interfaces associated with coexistence can be maintained, while the temperature naturally relaxes toward its coexistence value.^98^ If the system is initially too hot, partial melting absorbs latent heat and lowers the temperature; if it is too cold, the reverse occurs. Because the *NPH* ensemble allows unconstrained fluctuations in both temperature and volume at fixed pressure, it is particularly useful for locating equilibrium conditions and probing structural stability under (approximately) adiabatic evolution.^102,103^

Constant-*NPH* algorithms were originally introduced by Andersen^104^ and later benchmarked by Brown and Clarke^105^ against results obtained in other ensembles. Subsequent studies exploited this framework, in combination with direct coexistence, to determine the melting temperature of ice Ih,^106,107^ and later extended it to additional ice polymorphs over broad pressure ranges.^108^ More recently, the methodology has also been adapted to predict hydrate dissociation conditions, including the dissociation line of methane hydrate.^102^ Overall, the *NPH* ensemble is particularly attractive for exploring phase transitions and destabilization processes in condensed phases, as it captures the intrinsic thermodynamic response of the system without imposing an external temperature-control mechanism.^72,100^

Experimental dissociation and phase-equilibrium data for HQ clathrates are extremely scarce. The available information is limited to a few systems, including Ar–HQ, Kr–HQ, Xe–HQ and CH_4_–HQ clathrates. For instance, a triphasic coexistence point has been reported for the Ar–HQ system at approximately 463 K and 70 atm,^66^ which lies reasonably close to the pressure range explored in the present work. At 100 bar, our simulations predict an equilibrium temperature of 452(2) K, which is in qualitative agreement with the reported value of approximately 463 K, despite the differences in pressure and the limited amount of information currently available in the literature. Most of the remaining published studies focus on occupancies, equilibrium constants and intercalation thermodynamics of Xe–HQ and Kr–HQ clathrates rather than on complete dissociation lines.^70,71^ Furthermore, the available measurements are generally restricted to pressures substantially lower than those investigated here. Therefore, direct quantitative comparison with the coexistence conditions calculated in this work remains limited.

The rest of this article is structured as follows: the Molecular Models, Simulation Details, and Methodology Validation sections describe the employed models, simulation protocols, and the validation strategy adopted in this work, respectively. The results and their discussion are presented in the Results section. Finally, the main conclusions are summarized in the Conclusions section.

## Molecular Models

In this study, all molecular interactions are described using the OPLS-AA (Optimized Potentials for Liquid Simulations–All Atom) force field.^109,110^ OPLS-AA–compatible parameterizations for both the HQ host and the guest species have been reported and evaluated previously. In particular, the HQ host model was originally proposed and documented by Comesaña *et al.* ^58^ and has since been extensively employed and validated.^61,65,111–113^ Retaining this host description here preserves consistency with earlier work and provides a benchmarked framework for direct, reproducible comparisons. Guest molecules are modeled either as neutral, single-site spherical particles (Ar and CH_4_) or as three-site representations (H_2_ and CO_2_). The mass, charge, *σ*, and Lennard–Jones *ϵ* parameters for each interaction site are summarized in Table 1.

**Table 1:** Force-Field Parameters Employed for the Guest Molecules^a^

Guest	Site	Description	Mass/a.m.u.	*σ*/nm	*ϵ*/kJ·mol^–1^	Charge/e
Ar	Ar	Argon atom	39.9480	0.3401	0.9786	0.0000
CH_4_	C	Methane interaction site	16.0430	0.3730	1.2301	0.0000
CO_2_	C	Carbon atom	12.0110	0.3500	0.2761	0.8200
CO_2_	O	Oxygen atom	15.9994	0.2900	0.5858	–0.4100
H_2_	M	Central virtual LJ site	0.0000	0.3038	0.2852	–0.9864
H_2_	H	Hydrogen nucleus	1.0080	0.0000	0.0000	0.4932

a Ar and CH_4_ were described using OPLS-AA-based models.^110^ CO_2_ and H_2_ were represented using molecular models previously developed for HQ clathrates, in which atomic charges were obtained from quantum-chemical calculations.^56,58^

Methane is modeled as a single Lennard–Jones (LJ) sphere. This approximation has been used in earlier studies and shown to be robust.^61,112^

The parameters and charge distributions employed for CO_2_ and H_2_ correspond to molecular models previously developed and validated specifically for HQ clathrate simulations.^56,58^

For H_2_, a more detailed description is employed using the three-site quadrupolar model previously proposed for hydroquinone clathrates.^56^ In this model, two mass-bearing sites representing the hydrogen nuclei are separated by 0.07414 nm, while a massless virtual site is located at the molecular center. Lennard–Jones interactions are assigned exclusively to this virtual site. The total molecular mass is distributed over the two hydrogen nuclei, which carry positive partial charges, whereas a compensating negative charge is assigned to the central virtual site. This construction preserves the overall neutrality of the molecule while reproducing its quadrupolar character.

Finally, CO_2_ is modeled as a rigid three-site molecule following the molecular model previously developed for HQ clathrates.^58^ The Lennard–Jones framework is based on OPLS-AA parameters, whereas the molecular geometry and atomic charges were obtained from quantum-chemical calculations using the CHELPG electrostatic-potential fitting procedure.^58^ Two oxygen sites carrying partial negative charges are located symmetrically around a central positively charged carbon atom, reproducing the characteristic quadrupole moment of CO_2_. Given the relevance of both species, employing physically realistic molecular representations is important for accurately describing guest–host interactions within the crystal lattice.

Cross Lennard–Jones interactions were calculated using the standard OPLS-AA combination rules implemented in GROMACS (comb-rule = 3), consistent with the force-field parameterization employed in this work.

## Simulation Details

Molecular dynamics simulations are carried out using the version 2021.1 of GROMACS in double precision format.^114–121^ Simulations were performed using Intel Xeon Ice Lake 8352 processors and an NVIDIA A100-PCIE-40GB GPU. Each calculation is executed using 32 cores together with GPU support.

We employ the direct-coexistence approach, in which a *β*-HQ clathrate slab (576 HQ molecules and 192 guest particles) is placed at the center of the simulation box and flanked by liquid hydroquinone (576 HQ molecules in total, i.e., 288 on each side). The liquid phase serves as an interfacial layer connecting the clathrate to an outer region of guest molecules in a supercritical state (1000 particles). Periodic boundary conditions replicate this arrangement, enabling sustained three-phase coexistence. The simulation box had dimensions of 3.25 × 2.81 × 29.55 nm^3^. This size is large enough to avoid the effects of finite size. The system was initially constructed with 5 nm layers of guest fluid at both ends of the box, followed by 5 nm layers of HQ fluid. The central region contained a *β*-HQ seed with an initial length of 9.55 nm along the longest dimension of the simulation box.

All three-phase coexistence simulations in the production stage are performed in the isobaric–isoenthalpic (*NPH*) ensemble. As recently emphasized by Weidmann *et al.* ^102^ preparing and maintaining stable three-phase coexistence is delicate and requires careful control of the simulation cell. We therefore adopt the protocol described below for system setup, equilibration, and production.

System preparation consists of sequential equilibration steps aimed at generating a stable starting configuration for the *NPH* production runs. We first equilibrate in the *NVT* ensemble for 10 ns using the leapfrog–Verlet integrator with a 1 fs time step. Temperature is controlled with the velocity-rescale (V-rescale) thermostat (with a coupling constant of 0.1 ps). We then switch to the *NPT* ensemble and equilibrate for 15 ns, using the same integrator and time step, to reach the target pressure and the corresponding equilibrium density. During this stage, pressure is controlled with an anisotropic Berendsen barostat with a coupling constant of 16 ps, while the temperature is maintained with the same V-rescale thermostat.

From the *NPT* trajectory, we select the configuration whose instantaneous temperature is closest to the target value and use it as the initial condition for the subsequent *NPH* production run. In the *NPH* ensemble, pressure is controlled using the anisotropic Parrinello–Rahman barostat^122,123^ with a coupling constant of 16 ps. The compressibility is specified anisotropically as 4.5 × 10^–8^, 4.5 × 10^–8^, and 4.5 × 10^–5^ bar^–1^ along the principal directions.

To prevent premature melting or crystallization during equilibration when a phase transition occurs before completion, positional restraints are applied to molecules belonging to the affected phase. These restraints are imposed on the C and O atoms of each HQ molecule in the seed, using a harmonic potential with an isotropic force constant of 1000 kJ mol^–1^ nm^–2^.

Production simulations are then performed in the *NPH* ensemble using an integration time step of 0.1 fs for a total of 5.0 × 10^7^ steps.

Periodic boundary conditions are applied in all three spatial directions. Long-range electrostatics are treated with particle-mesh Ewald (PME)^124,125^ using interpolation order 4, a mesh spacing of 0.2 nm, and a real/reciprocal-space tolerance of 10^–5^. The Coulomb and Lennard–Jones cutoffs are set to 1.3 nm, and standard long-range dispersion corrections are applied to the energy and pressure. Bond constraints are enforced with LINCS (order 8, two iterations). A Verlet cutoff scheme is used with a buffer tolerance of 9.0 × 10^–8^.

Finally, except for the reduced time step in the production stage, all simulation settings follow those of Weidmann *et al.*.^102^ We found that using a larger time step prevents the enthalpy from converging to a constant value in the *NPH* runs.

## Methodology Validation

As noted above, it is important to emphasize that this type of analysis lacks direct experimental benchmarks against which the results can be validated or compared. We also stress that, to the best of our knowledge, this is the first application of the *NPH* ensemble to an HQ clathrate system. Nonetheless, Weidmann *et al.* ^102^ recently employed *NPH* simulations to determine the three-phase dissociation temperature (*T*_3_) of a clathrate system, specifically CH_4_ hydrate, obtaining excellent agreement with literature values. Although HQ clathrates have a more complex crystal structure than methane hydrate, the underlying coexistence problem is closely related, and there is no a priori reason why the same methodology should not be applicable to clathrate systems.

However, before applying this methodology to the HQ clathrate systems considered here, we have first validated it on a well-characterized clathrate-forming system. Specifically, we compute the three-phase dissociation temperature *T*_3_ of CO_2_ hydrate at 400 bar. This choice is motivated by the extensive simulation literature reporting *T*_3_ for CO_2_ hydrates, which provides a solid basis for comparison.^75,78,83,84,94^ In particular, some of us have previously determined *T*_3_ at 400 bar using the TIP4P/Ice water model and the TraPPE CO_2_ model.^126,127^ In those studies, *T*_3_ was obtained via two independent routes – direct coexistence and the solubility method – and both approaches agreed within statistical uncertainty, yielding *T*_3_ = 290(2) K at 400 bar.^84,94^ We therefore use this well-established reference value as a benchmark to assess the reliability of the *NPH*-based protocol.

The initial CO_2_ hydrate configuration was constructed by replicating the hydrate unit cell three times along each Cartesian direction, yielding a hydrate slab containing 1242 water molecules and 216 CO_2_ molecules. Pure liquid water (1242 molecules) and pure liquid CO_2_ (400 molecules) were then appended along the *z* direction, producing a three-phase setup (CO_2_ hydrate/liquid water/liquid CO_2_) with interfaces normal to *z*. Simulation settings closely follow those used for the HQ clathrate, with minor differences: the hydrate system requires shorter equilibration times, a different cutoff, and a specific treatment of water–CO_2_ cross interactions. Here we employ a 1.6 nm cutoff, and compute dispersion cross interactions using a modified Berthelot rule in which the Lennard–Jones parameter is *ϵ*_12_ = *ξ*(*ϵ*_11_ *ϵ*_22_)^1/2^, with *ξ* = 1.13. This choice ensures consistency with our previous studies^84,94^ where *T*_3_ for CO_2_ hydrate was obtained via two independent methods. Using identical interaction definitions should yield the same *T*_3_ even when a different computational protocol is adopted.

After constructing the initial simulation cell and defining the interactions to match our previous work, we performed two short equilibration runs in the *NVT* and *NPT* ensembles to bring the system to the target pressure (400 bar) and the desired temperatures. Four initial temperatures were considered in this study: 270, 280, 290, and 295 K. Each equilibration stage lasted 4 ns. All settings were the same as those used for the clathrate system, except for the cutoff value and the modified Berthelot rule described above. Production simulations were then performed in the *NPH* ensemble for 100 ns, using the same protocol as for the HQ clathrate.

As shown in Figure 1 (left panel), the temperature trajectories converge to a common value after 75 ns, approximately, regardless of the initial equilibration temperature. Averaging the four independent runs over the 75–100 ns interval and computing the corresponding standard deviation yields *T*_3_ = 289.2(1) K. This value is in excellent agreement with the previously reported *T*_3_ = 290(2) K obtained using the same interaction model but determined via direct coexistence^84^ and the solubility method.^94^ Figure 1 (right panel) shows the enthalpy as a function of time. In all cases the enthalpy remains essentially constant, except for the highest initial temperature, which exhibits a step-like change. This feature reflects partial melting of the hydrate slab when the starting temperature exceeds *T*_3_; the system then relaxes toward its coexistence state. Importantly, both temperature and enthalpy are stable over 75–100 ns, the window used to compute *T*_3_. These results support the transferability of the *NPH*-based protocol introduced by Weidmann *et al.* ^102^ from CH_4_ hydrate to other hydrate-like systems.

**Figure 1:**
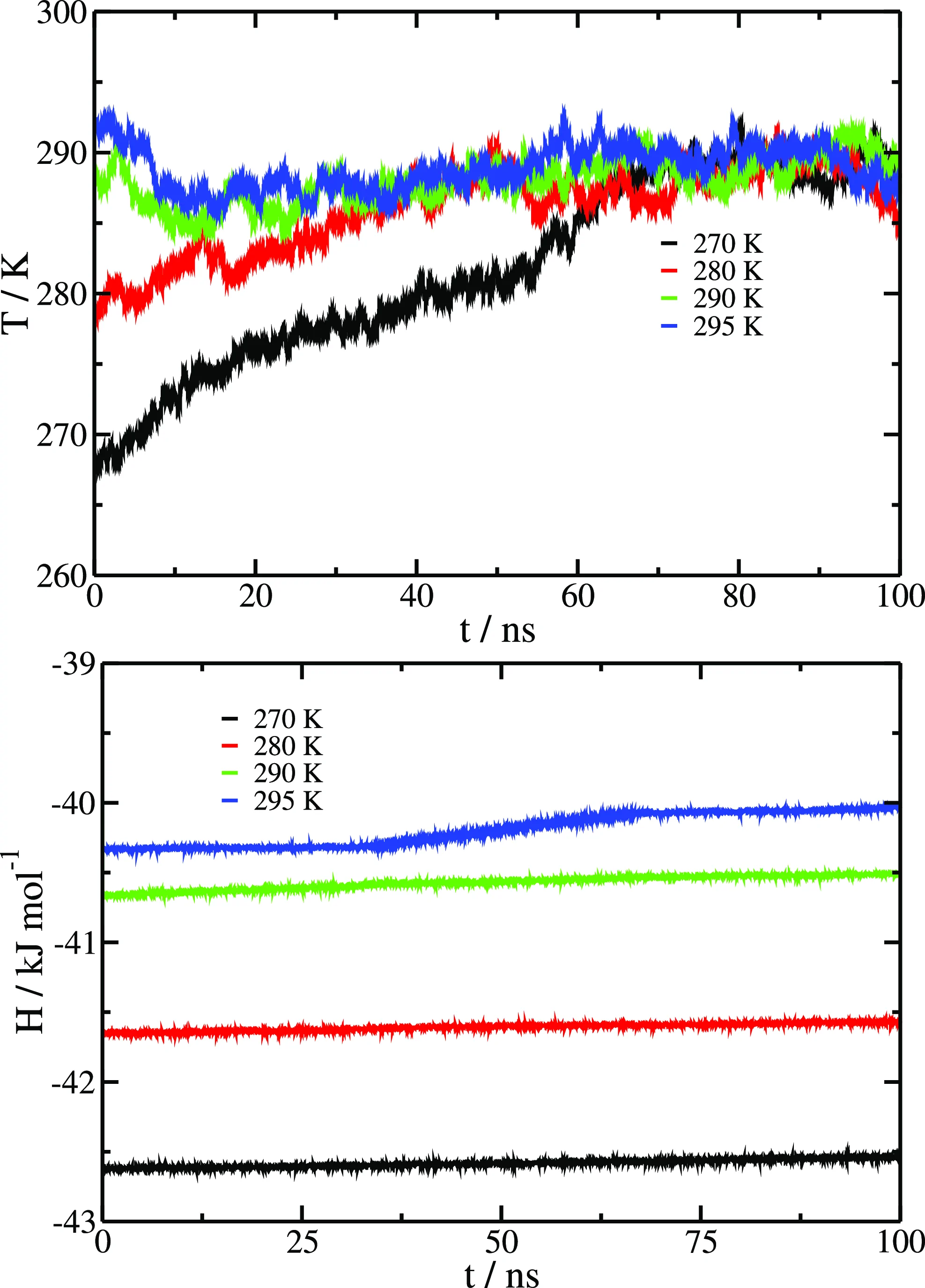
Temporal evolution of temperature (left) and enthalpy (right) in CO_2_ hydrate *NPH* simulations. In the temperature temporal evolution, a 500-step moving average is applied.

## Results

This section presents the results of the *NPH* simulations performed for the HQ clathrate with the aforementioned guests: Ar, CH_4_, H_2_, and CO_2_. The analysis includes the temporal evolution of temperature in independent replicates under different initial conditions, as well as the determination of the equilibrium temperature for each guest in a pressure range between 100 and 1000 bar. These data allow us to evaluate the influence of pressure and the nature of the guest on the stability of the system and its thermal behavior.

All simulated systems have a three-phase configuration, consisting of a *β*-hydroquinone crystal seed in the center, flanked by a liquid phase of hydroquinone that acts as an interfacial region connecting an outer layer of guest molecules in a supercritical state. This arrangement allows the interaction between the crystal lattice and the fluid to be studied under extreme conditions. However, in the case of carbon dioxide, this three-phase separation is not maintained: CO_2_ dissolves in hydroquinone or, alternatively, hydroquinone solubilizes in CO_2_, giving rise to a two-phase system instead of the three-phase scheme observed with the other guests. Consequently, the equilibrium reached in the CO_2_ simulations differs fundamentally from that obtained for Ar, H_2_, and CH_4_. The temperatures reported for CO_2_ therefore correspond to a clathrate–solution coexistence equilibrium rather than to a three-phase dissociation temperature. For this reason, the CO_2_ values should be interpreted separately and should not be compared quantitatively on an equal footing with the three-phase coexistence temperatures obtained for the other guest species.

An example of an initial configuration is shown in the Figure 2a, corresponding to an HQ system with H_2_ as guest. If this configuration is subjected to equilibration prior to the process in the *NPH* ensemble at a temperature lower than the unknown equilibrium temperature, the result will be partial crystallization. This is illustrated in the Figure 2b. Similarly, the Figure 2c shows a configuration with partial melting of the crystalline phase, which corresponds to a replica in which the equilibration was performed at a temperature higher than the equilibrium temperature. It should be noted that these configurations cannot correspond to equilibrium; otherwise, the phase change process would be total, and the system would become biphasic, or monophasic in the case of CO_2_ as a guest.

**Figure 2:**
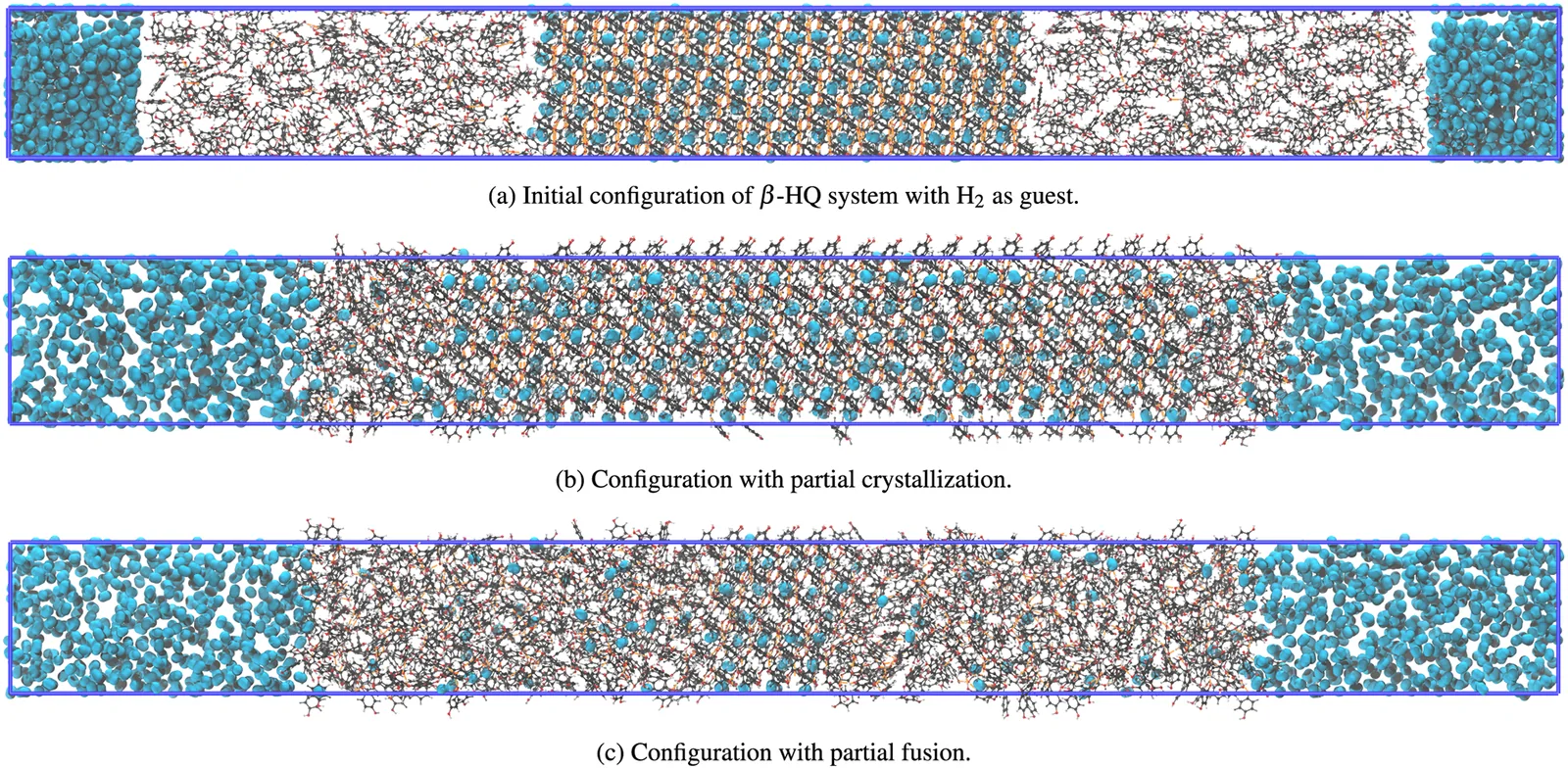
Visual representation of the initial configuration (a), the equilibrium with partial crystallization (b), and the equilibrium with partial fusion (c) of a *β*-HQ system with H_2_ as the guest. Molecular hydrogen atoms are represented by cyan spheres, while those belonging to HQ are shown by white spheres. The carbon skeleton is shown by black sticks and spheres. Oxygen atoms are shown in red, and hydrogen bonds are represented by orange dashed lines.

The evolution of temperature (T) as a function of time (t) for four different simulations is shown in Figure 3a. These correspond to a system with H_2_ as guest at 750 bar, with initial temperatures of 440 K (black), 450 K (red), 460 K (green) and 470 K (blue). All replicas converge in the same range at equilibrium after an initial adjustment period lasting approximately 28 ns. This can be better appreciated in the Figure 3b, which shows the same information but applies smoothing using a moving average with a window of 1000 steps. From that point on, the temperature fluctuations are characteristic of the *NPH* ensemble. In these, rapid oscillations around the mean value are observed due to the absence of a thermostat. The superimposition of the curves indicates that the system reaches a consistent thermodynamic state, independent of the initial temperature, confirming the stability of the simulation protocol under the imposed conditions.

**Figure 3:**
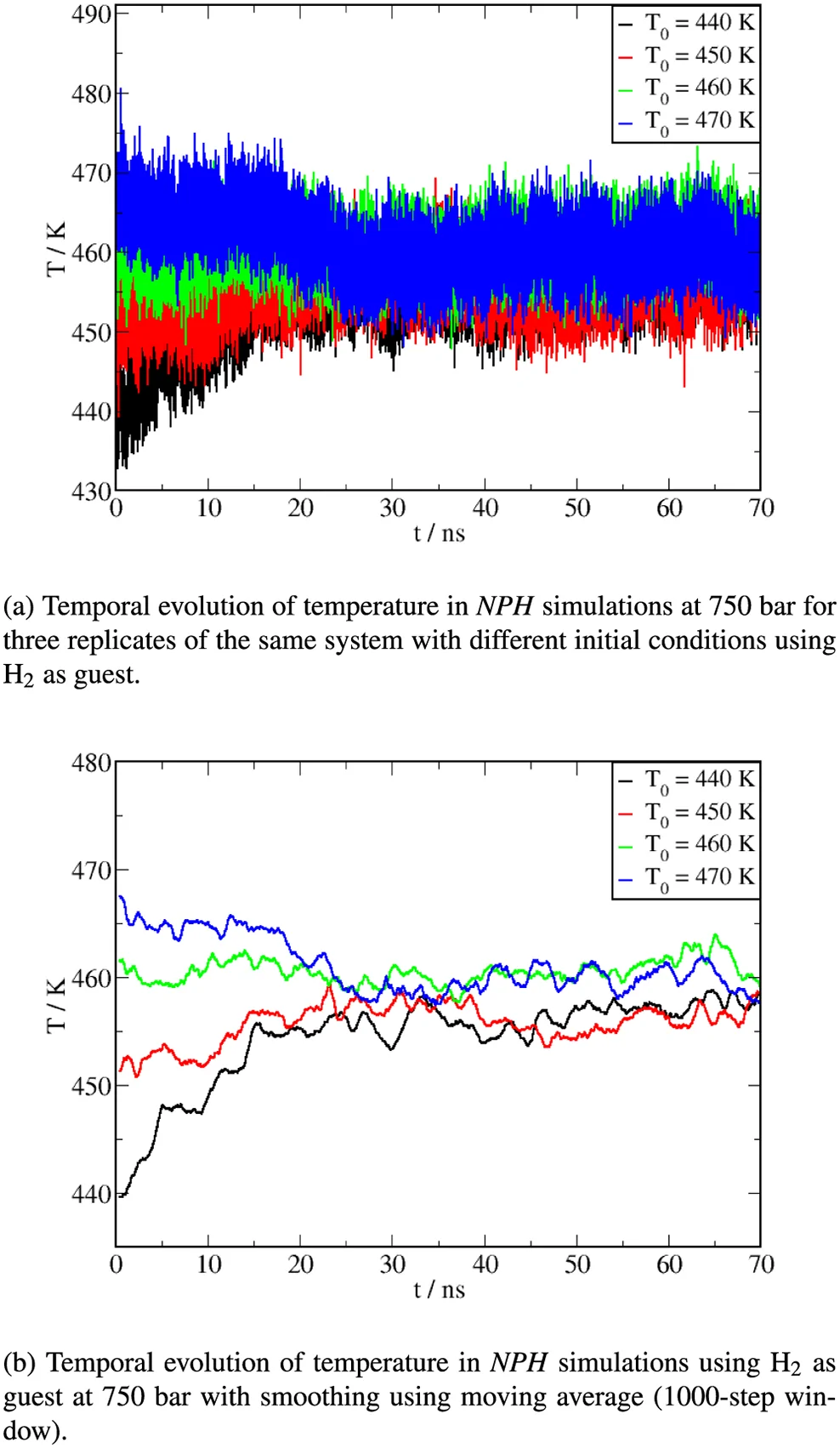
Temperature evolution in *NPH* simulations at 750 bar using H_2_ as guest: (a) raw trajectories for three replicates; (b) smoothed trajectories (1000-step moving average).

To estimate the average temperature and its uncertainty, a block analysis was used. Once the replicas have converged to equilibrium, the remaining interval is divided into a fixed number of blocks (by default, 15), calculating the average temperature in each one. This approach reduces the temporal correlation between individual microstates and provides a more realistic estimate of the statistical error. For each replica, the classical statistics for block averaging are then applied. Finally, an equally weighted summary is obtained that includes the global mean and standard deviation, considering all the replicates analyzed. This method is suitable for *NPH* simulations, where fluctuations are significant and independence between configurations cannot be assumed.

The Figure 4 shows an example of the variation in molar enthalpy (H) as a function of time for an HQ system with H_2_ as a guest at 500 bar and an initial temperature of 440, 450, 460, and 470 K. Otherwise, complete melting or absolute crystallization of the fluid HQ phase would occur, which would be contrary to the initial intention of studying a three-phase mixture. Therefore, when the thermostat of a system in a state far from equilibrium is disconnected, the system exhibits fluctuations characteristic of the *NPH* ensemble.

**Figure 4:**
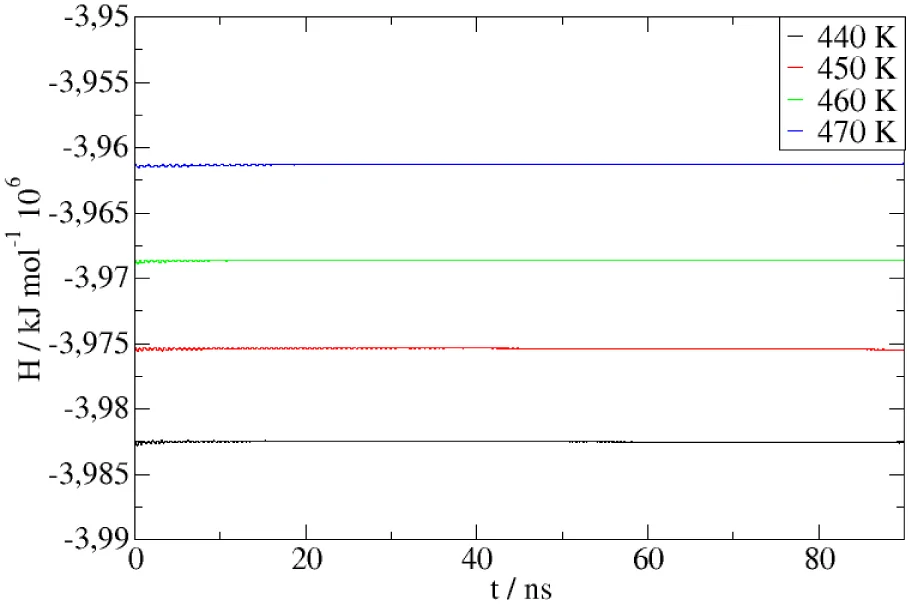
Temporal evolution of the system enthalpy as a function of time during the simulation for the system with H_2_ as the guest at 500 bar and 440, 450, 460, and 470 K.

The same procedure described for H_2_ at 1000 bar has been applied to the other proposed species. The Table 2 shows the average temperature reached by the system after the stabilization phase, together with the statistical uncertainty (in parentheses), for the four guests. The graphical representation of the values given by this is shown in Figure 5. A general trend of increasing temperature with pressure is observed, consistent with the compressibility of the system and the thermal response under *NPH* conditions. The effect is more pronounced for nonpolar guests (Ar and CH_4_), which show increases of approximately 40 K between the maximum and minimum values proposed for pressure, while for smaller molecules or those with specific interactions (H_2_ and CO_2_), the increase is more moderate. This reflects substantial differences in structural stability caused by guest-hydroquinone interaction and the adjustment of each atom or molecule to the cell in which it is contained.

**Table 2:** Equilibrium Temperature (K) Obtained in *NPH* Simulations for HQ with Different Guests and Pressures^a^

Guest	100 bar	500 bar	750 bar	1000 bar
Ar	452(2) K	475(1) K	483.8(7) K	489.4(7) K
CH_4_	460(4) K	483(3) K	492.0(8) K	500(2) K
H_2_	440.4(6) K	453(2) K	458(2) K	465(2) K
CO_2_	440(1) K	444(2) K	444(2) K	452.2(5) K

a The CO2 entries should be interpreted separately from the three-phase coexistence temperatures of the other guests, as they correspond to those associated with a two-phase system.

**Figure 5:**
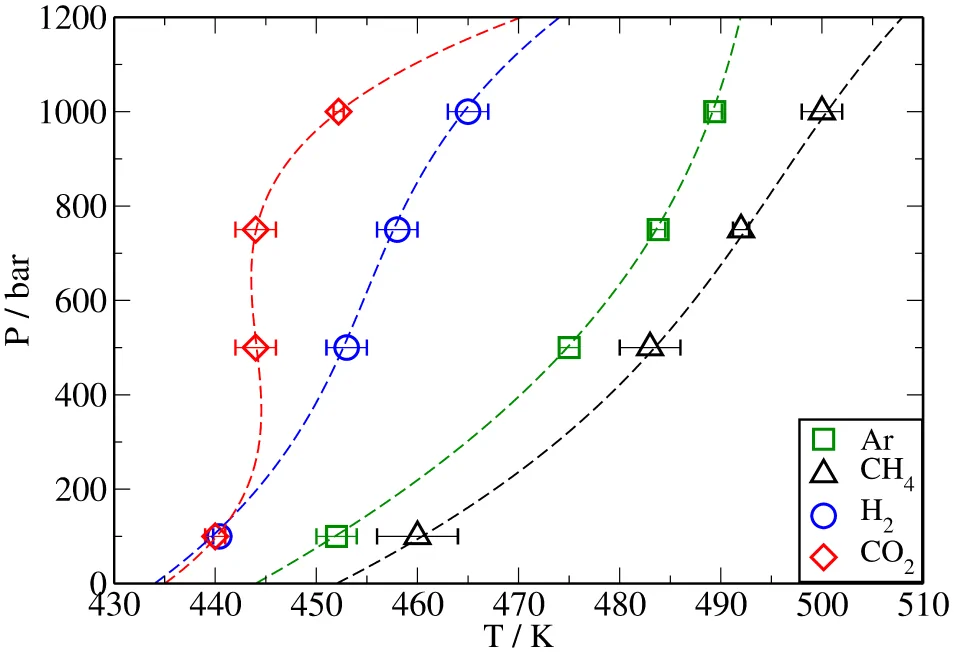
Representation of the different equilibrium temperatures calculated in K for each of the different guests included in the system against the pressure fixed in bar. The data correspond to Ar, CH_4_, H_2_ and CO_2_, represented respectively by green squares, black triangles, blue circles, and red diamonds. The horizontal bars indicate the uncertainty in temperature.

In general, guests with a size closer to that of the crystalline cells in which they are contained provide the system with greater structural stability. The packing influences the long-range diffusion energy within the crystal lattice, as a denser structure increases the energy barriers to the movement of guest molecules, modulating both the dynamics and accessibility of the internal channels.^61,112^

For all the reasons mentioned above, the results obtained from simulations in the *NPH* ensemble are considered robust and logical, which strengthens the viability of the method for characterizing the thermal stability of HQ clathrates under a wide range of conditions. The data presented in this work provide a quantitative basis for understanding the stability mechanisms in these systems and constitute a starting point for evaluating their potential in gas storage and separation applications.

## Conclusions

This study determined the clathrate stability limit of *β*-HQ systems with different guests over a pressure range of 100 and 1000 bar. To this end, molecular models based on the OPLS-AA force field were used, which aim to realistically and reliably emulate the behavior of various real guests: Ar, CH_4_, H_2_, and CO_2_. The system was prepared by equilibration steps in *NVT* and *NPT* ensembles prior to production in *NPH*. Periodic conditions, pressure control with the Parrinello–Rahman barostat, electrostatic treatment using PME, and constraints with LINCS were implemented, all executed in GROMACS 2021.1 in double precision.

The analysis focused on the temporal evolution of temperature and enthalpy. The coexistence temperature was estimated using the statistical block method of fluctuations from several replicates with different initial temperatures after reaching equilibrium. This approach constitutes not only the first application of the *NPH* ensemble to organic clathrates, providing a pioneering methodology for systems with high structural reorganization, but also one of the first approaches to determine the thermodynamic stability conditions of these clathrate systems.

It was observed that the system reaches a thermodynamic state that is independent of the initial conditions at a given pressure. The general trend indicates that the equilibrium temperature increases with pressure, reflecting the compressive response of the crystal in its structural stability. On the other hand, significant differences were identified between the different guests analyzed. Large, nonpolar gases such as argon and methane show more pronounced increases in equilibrium temperature with pressure, while small species or those with specific interactions, such as H_2_ and CO_2_, show more moderate variations. This behavior is consistent with expectations and shows that the nature of the guest affects the compactness of the crystal and, therefore, the internal dynamics and accessibility of the channels.

This research demonstrates that the use of the *NPH* ensemble is an effective and robust tool for exploring the conditions of formation and destabilization in systems with high structural organization, such as organic clathrates. The absence of experimental references makes this study a pioneering contribution, which not only provides data on coexistence conditions, but also provides a basis for future studies for the design of materials aimed at storing gases, especially H_2_, in safe and reversible environments.

## Data Availability

The data that support the findings of this study are available within the article.
